# Sulfamethoxazole Leaching from Manure-Amended Sandy Loam Soil as Affected by the Application of Jujube Wood Waste-Derived Biochar

**DOI:** 10.3390/molecules26154674

**Published:** 2021-08-02

**Authors:** Mohammad I. Al-Wabel, Munir Ahmad, Muhammad I. Rafique, Mutair A. Akanji, Adel R. A. Usman, Abdullah S. F. Al-Farraj

**Affiliations:** 1 Soil Sciences Department, College of Food & Agricultural Sciences, King Saud University, P.O. Box 2460, Riyadh 11451, Saudi Arabia; amunir@ksu.edu.sa (M.A.); mrafique@ksu.edu.sa (M.I.R.); mtex555@gmail.com (M.A.A.); adosman@ksu.edu.sa (A.R.A.U.); sfarraj@ksu.edu.sa (A.S.F.A.-F.); 2 Department of Soils and Water, Faculty of Agriculture, Assiut University, Assiut 71526, Egypt

**Keywords:** vertical translocation, release dynamic, leaching kinetics, retention, groundwater contamination

## Abstract

Vertical translocation/leaching of sulfamethoxazole (SMZ) through manure-amended sandy loam soil and significance of biochar application on SMZ retention were investigated in this study. Soil was filled in columns and amended with manure spiked with 13.75 mg kg^−1^ (S1), 27.5 mg kg^−1^ (S2), and 55 mg kg^−1^ (S3) of SMZ. Jujube (*Ziziphus jujube* L.) wood waste was transformed into biochar and mixed with S3 at 0.5% (S3-B1), 1.0% (S3-B2), and 2.0% (S3-B3) ratio. Cumulative SMZ leaching was lowest at pH 3.0, which increased by 16% and 34% at pH 5.0 and 7.0, respectively. A quicker release and translocation of SMZ from manure occurred during the initial 40 h, which gradually reduced over time. Intraparticle diffusion and Elovich kinetic models were the best fitted to leaching data. S3 exhibited the highest release and vertical translocation of SMZ, followed by S2, and S1; however, SMZ leaching was reduced by more than twofold in S3-B3. At pH 3.0, 2.0% biochar resulted in 99% reduction in SMZ leaching within 72 h, while 1.0% and 0.5% biochar applications reduced SMZ leaching to 99% within 120 and 144 h, respectively, in S3. The higher SMZ retention onto biochar could be due to electrostatic interactions, H-bonding, and π-π electron donor acceptor interactions.

## 1. Introduction

Antibiotics are generally recommended for treatment of some infective diseases in humans and animals. Worldwide, total production and consumption of antibiotics has surpassed 200,000 tons annually, and is estimated to increase by up to 200% by 2030 [1]. Veterinary antibiotics (VAs) are being used extensively in livestock industry with purpose to increase animal growth, meat and milk production and for prevention of infectious diseases in farm animals. These VAs are partially consumed by animals and are excreted into the environment through their feces and urine. Consequently, dumping of such livestock waste and animal manure on soil or its application as organic fertilizer potentially results in accumulation of such VAs in soil and thus pollute the terrestrial and aquatic ecosystem.

Tetracyclines, penicillin and sulfonamides groups are the most commonly and invasively consumed VAs worldwide [2]. Sulfamethoxazole (SMZ), which belongs to sulfonamides antibiotic group has been extensively consumed since the 1960’s for treatment of human and animal diseases. Sulfonamides are regularly used in cattle, poultry feed and drinking water at different rates to improve chick growth, animal health and meat production [3]. SMZ is not fully metabolized in animal’s body and 50–100% of it can be released out of the body through manure and urine [4]. Therefore, it has frequently been detected in waste dumping areas, water resources, soil, and livestock manure [5]. Higher amounts of SMZ residues were found in swine-manure-amended soil [6]. In separate studies, Boxall et al. [7] and Gobel et al. [8] found SMZ contents in cattle-manure-fertilized soil. Likewise, Wu et al. [9] reported the development of soil pollution, as well as production and growth of antibiotic resistance bacteria and pathogens by entrance of certain antibiotics including SMZ in soil by employing livestock manure and sewage sludge.

Previous studies reported relatively higher mobility of SMZ in soil and which is a potential risk for groundwater contamination [10]. Once added to soil, antibiotics pass through a number of chemical, biological and structural degradation processes including adsorption, retention, transport, runoff, leaching and plant uptake which lead to pervasive environmental pollution. Hill et al. [11] studied the leaching of sulfonamides (including SMZ) in a column experiment and found that a higher proportion of sulfonamides leached down with irrigation. Additionally, working on SMZ adsorption in organic soil, Chen et al. [12] reported a higher leaching of SMZ due to its higher solubility, which could subsequently pollute ground and surface water resources. For instance, Luo et al. [13] and Carballa et al. [14] detected higher concentrations of SMZ in wastewater (940 ng L^−1^ in China and 164–1700 ng L^−1^ in Europe). Hence, SMZ antibiotics have been deemed a potential organic pollutant globally, and, therefore, it is very important to develop remediation strategies to mitigate SMZ toxicity in aquatic and terrestrial ecosystems. A number of versatile remediation process and techniques have been employed to restrict antibiotics release into soil and water resources such as biological and chemical treatments, electrokinetic remediation, advanced oxidation methods, membrane separation, activated carbon, organic filters, and adsorption [15]. However, the majority of these techniques are either expensive or inefficient. Therefore, there is a dire need to develop cost-effective, greener, and eco-friendly approaches to decrease the translocation of SMZ residues in soil, into nearby and underground water resources. 

Biochar (BC), which is a byproduct of controlled combustion of organic waste [16] has gained attention as a promising adsorbent to remove organic and inorganic pollutants from soil and waste water resources [15,17]. Employing BC as soil amendment for removing pollutants has been considered more effective and auspicious approach in comparison with other conventional techniques. Diverse surface properties, higher surface area, porosity, several surface functional groups, ample carbon contents, and surface charge make it an apposite adsorbent for a lot of organic and inorganic pollutants [18,19,20]. Therefore, if applied in contaminated soil, BC may reduce the mobility/leaching of pollutants on one hand, and improve soil physiochemical properties on the other hand. However, limited research is available in the literature regarding the application of jujube (*Ziziphus jujube* L.) waste-derived BC for antibiotics retention in soil and to reduce its translocation into nearby and underground water resources. Therefore, the current study was conducted to investigate the vertical translocation behavior of SMZ in a sandy loam soil amended with SMZ-laden manure under different pH levels in column leaching trials. Moreover, the significance of jujube wood waste derived BC in enhancing the retention of SMZ in contaminated-manure-amended sandy loam soil was also explored. 

## 2. Results and Discussion

### 2.1. Characterization of Soil, Manure, and Biochar

**Figure 1:**
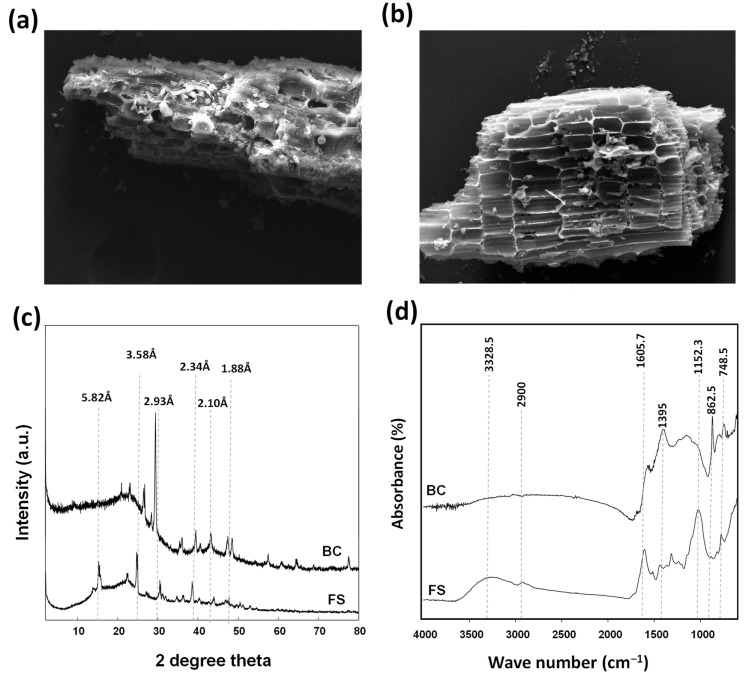
(**a**) Scanning electron microscope images of jujube wood waste (FS), (**b**) scanning electron microscope images of jujube wood waste-derived biochar (BC) at magnification of 800 X, (**c**) X-ray diffraction analyses patterns of FS and BC, and (**d**) Fourier transform infrared spectroscopy spectra of FS and BC.

Physicochemical characterizations of soil, manure, and BC are shown in Table 1. The soil used in the study was sandy loam in texture with slight alkaline pH and electrical conductivity (EC) of 2.41 dS m^−1^. Soil showed lower organic matter contents (0.62%) and calcareousness (CaCO_3_ = 8.45%). The lower available phosphorus (P) contents in soil (9.16 mg kg^−1^) could be due to higher CaCO_3_ contents in soil which might have resulted in P fixation and its reduced bioavailability. The contents of nitrate in soil were very low (0.88 mg kg^−1^), while K and Na were 78.00 and 25.81 mg kg^−1^, respectively. The cation exchange capacity (CEC) of soil was 21.19 cmol kg^−1^, while that of manure was 57.29 cmol kg^−1^. The pH of the collected manure was 7.72 and EC was 6.61 dS m^−1^. Higher contents of P and nitrate were seen in the manure sample (1970.82 and 13.90 mg kg^−1^). Freshly produced BC was analyzed for its physiochemical characteristics as well. A significant increase in pH of BC against feedstock (FS) was observed, which could be due to decomposition of organic compounds by thermal treatment of FS which consequently raised removal and release of acidic functional groups and alkali salts, respectively [21,22]. In contrast to pH, a decline in CEC value of BC was observed, which might be due to thermal degradation and removal of organic components and surface functional groups such as carboxylic, hydroxyl and oxygen containing functional groups [23]. Surface functional groups degradation and removal was also supported by Fourier transformation infrared (FTIR) analysis of produced material (Figure 1d). Controlled thermal combustion of FS caused thermal breakdown of cellulose and hemicellulose contents in FS and produced 36.7% of solid BC. The manure, FS, and BC samples were analyzed for proximate analyses. Moisture and volatile matter contents in BC were about two times less than FS, which might be due to dehydration of organic compounds in FS by pyrolysis [24]. This can also be correlated with moisture analyses given in Table 1, showing higher contents of moisture in FS than in BC. Produced BC showed noticeably higher ash contents (11.02%) than raw FS (4.67%), which are mainly attributed to formation and condensation of mineral compounds in BC [25]. Carbonization of raw FS resulted in considerably higher carbon contents in BC which showed higher recalcitrant potential, carbon stability and aromaticity of BC over FS. A similar trend of BC characterization was found in previous studies conducted by Rafique et al. [19,20]. The moisture contents of manure were relatively higher (13.03%), while volatiles, fixed carbon, and ash contents were in lower range of 38.72%, 19.29%, and 28.96%, respectively. Morphological analyses of raw FS and its derived BC showed that porous surface of BC, which was mainly attributed to the loss of volatiles and organic compounds as affected by heat treatment (Figure 1a,b). Produced BC possessed smaller and more pores, while the FS was characterized by irregular pores on its surface which might be due to higher removal of volatile compounds in FS and BC, respectively. The XRD spectra indicated mineralogical differences between FS and BC (Figure 1c). The peaks appearing at 3.58 Å and 5.82 Å depicted the presence of whewellite (CaC_2_O_4_·H_2_O) and cellulose compounds in FS, which latterly reduced in BC. The peaks at 2.93 Å, 2.10 Å, and 1.88 Å were designated as calcite, which were intense in BC [26,27]. The peaks appearing at 2.34 Å in both FS and BC were attributed to sylvite. The FTIR spectra showed the bands of O–H around 3328.5 cm^−1^, indicating H-bonding associated with water molecules, while the bands around 2900 cm^−1^ were designated as stretching of C–H groups, which were absent in BC (Figure 1d). The stretching of carboxylic groups (–COOH) was observed around 1605.7 cm^−1^ in FS, which reduced in BC due to thermalization. The bands appearing around 862.5 and 1395 cm^−1^ were attributed to the stretching of C–O, while bands around 748.5 cm^−1^ were designated as Si–O [28]. A band with higher intensity in FS around 1152.3 cm^−1^ was attributed to C–O–C stretching of polysaccharide cellulose, which disappeared in BC [15]. Similar results were also observed in XRD patterns, where FS exhibited relatively higher contents of cellulose and hemicellulose, which disappeared in BC with thermalization. Thus, a nearly similar trend of appearing and declining of volatiles and organic compound (cellulose, hemicellulose) peaks in FS and BC due to thermal treatment was observed in both XRD and FTIR analyses. Therefore, these results indicated the O-containing functional groups were dominant on the surface of the produced biochar. These functional groups were C–O, –COOH, C–O–C, and Si–O. 

### 2.2. Effect of pH on SMZ Leaching 

**Figure 2:**
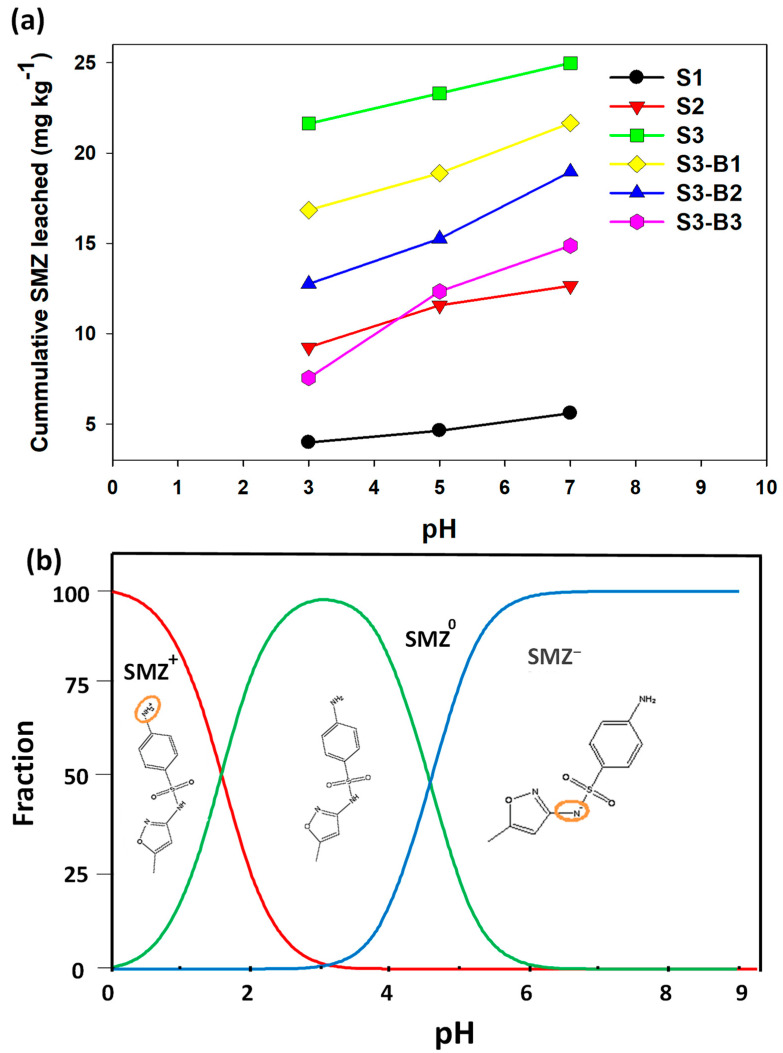
(**a**) Effects of pH on sulfamethoxazole (SMZ) leaching from various contaminated manure-amended soils with and without biochar applications (S1: soil with manure containing 13.75 mg kg^−1^ of SMZ, S2: soil with manure containing 27.5 mg kg^−1^ of SMZ, S3: soil with manure containing 55 mg kg^−1^ of SMZ, S3-B1: treatment S3 with 0.5% biochar application, S3-B2: treatment S3 with 1.0% biochar application, S3-B3: treatment S3 with 2.0% biochar application) and (**b**) transformation of SMZ forms with changing the pH.

The effects of background solution pH on SMZ leaching from soil amended with contaminated manure after BC application are demonstrated in Figure 2. Soil (S) was amended with spiked-manure at three different rates of 13.75 (S1), 27.5 (S2), and 55 mg kg^−1^ (S3). Further S3 was amended with BC at three different rates i.e., 0.5%, 1.0%, and 2.0% (S3-B1, S3-B2, and S3-B3, respectively). The results showed an overall increase in cumulative SMZ leaching with increase in pH from 3.0 to 7.0 (Figure 2a). An increase in pH from 3.0 to 5.0 resulted in average increase in cumulative leaching by 16%, while the increase in pH from 3.0 to 7.0 leached on average 34% more SMZ cumulatively. However, this trend was different in S3-B3, where increase in pH from 3.0 to 5.0 and 7.0 resulted in 63% and 97% higher SMZ leaching, respectively. Soil S1 showed the lowest cumulative SMZ leaching, as it was amended with the manure containing the lowest amount of SMZ (13.75 mg kg^−1^). The highest SMZ cumulative leaching was observed in S3 soil, which was amended with the manure containing the highest SMZ concentration (55 mg kg^−1^); however, when amended with BC, the SMZ leaching was reduced. Interestingly, the soil treated with BC showed substantially higher SMZ leaching after increasing the pH from 3.0 through 7.0. For instance, soil S3 exhibited 7.7% and 15.5% higher SMZ leaching when pH was increased from 3.0 to 5.0 and 7.0, respectively; however, with 0.5%, 1.0% and 2.0% BC application, this increase was 12.1% and 28.6%, 19.7% and 48.6%, and 63.4% and 97.0%, respectively. 

Variations in the cumulative SMZ leaching with changing the pH was due to different speciation of SMZ at different pH levels along with the physiochemical properties of the soil [29]. It has previously been reported that SMZ is an amphoteric compound, which changes its speciation depending upon the pH of the medium (Figure 2b). The basic amine group (–NH_2_) is responsible for protonation, while the acidic sulfonamide group (–SO_2_NH–) is responsible for ionization [30]. Therefore, with changing the pH, the SMZ could behave either as a cation, anion, or neutral ion. At pH 1.0, SMZ is predominantly positively charged (SMZ^+^), which changes to neutral (SMZ^0^: zwitterion) when the pH goes beyond 4.0, and negative (SMZ^−^) at pH > 7.0. Therefore, the lower leaching at pH 3.0 was due to electrostatic attraction between cationic SMZ species and negatively charged surfaces of either soil or BC. However, with an increase in pH in the environment, the positive charges on the SMZ start transforming into neutral and subsequently negative charges. Therefore, in pH range of 4.0–5.0, the neutral SMZ species were the most dominant, which can be adsorbed onto soil or BC via H-bonding, hydrophobic distribution, and van der Waals attraction. However, as the pH goes beyond 7.0, the electrostatic repulsion between negatively charged SMZ and negatively charged soil/BC surfaces results in reduced adsorption and enhanced leaching [31]. Even at pH above 7.0, some portion of SMZ^−^ can be adsorbed onto soil particles via a bridging effect of Ca^2+^ [32]. A reduced adsorption of SMZ onto soil has also been reported previously [33]. For instance, Białk-Bielińska et al. [34] observed negative correlation between pH and K_d_ (sorption coefficient) in pH interval of 5.27–7.38 for sulfonamide compounds, indicating a decline in their adsorption at higher pH levels. Therefore, the higher adsorption of SMZ at pH 3.0 resulted in lower cumulative leaching, whereas the lower adsorption at pH 5.0–7.0 was due to repulsive forces, which subsequently enhanced the cumulative SMZ leaching [35]. However, the addition of BC resulted in lower leaching of SMZ as compared to the same treatment without BC at specific pH levels, suggesting the involvement of other mechanisms in SMZ adsorption onto BC besides electrostatic interactions. Yang et al. [36] suggested that the organic components in BC could result in SMZ adsorption onto BC through π-π electron-donor-acceptor (EDA) interactions and hydrophobic interactions. Therefore, the cumulative leaching and retention of SMZ in sandy loam was dependent on pH of the media, as well as the presence/absence of BC.

### 2.3. Temporal Leaching of SMZ 

**Figure 3:**
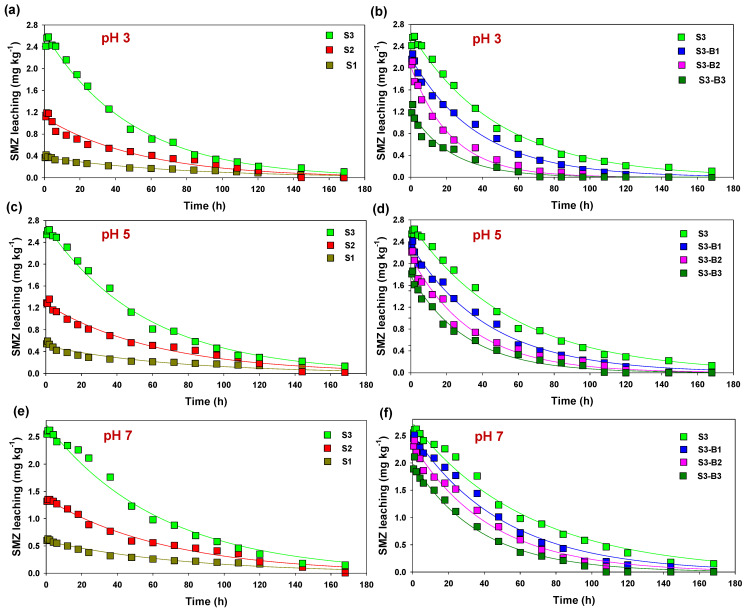
Leaching dynamics of sulfamethoxazole (SMZ) from various contaminated manure-amended soils with and without biochar applications at pH of 3.0 (**a**,**b**), 5.0, (**c**,**d**) and 7.0 (**e**,**f**). (S1: soil with manure containing 13.75 mg kg^−1^ of SMZ, S2: soil with manure containing 27.5 mg kg^−1^ of SMZ, S3: soil with manure containing 55 mg kg^−1^ of SMZ, S3-B1: treatment S3 with 0.5% biochar application, S3-B2: treatment S3 with 1.0% biochar application, S3-B3: treatment S3 with 2.0% biochar application).

The transport of SMZ through contaminated-manure-amended soil with and without BC application is shown in Figure 3. To present the results in a more understandable way, the treatments were divided in two groups, i.e., with BC application and without BC application at different pH levels (Figure 3). Overall, the higher concentrations of SMZ were present in leachates in the beginning, which slowly decreased over time. More specifically, the SMZ leaching increased from 0.5 to 1.0 h, deceased quickly from 1.0 to 40 h, and then approached slowly towards equilibrium. The highest SMZ leaching in the beginning was due to quick desorption from spiked manure. As the time progressed, the freely bound SMZ molecules were dissolved and leached, and finally reached a minimum level. The leaching and retention of sulfonamide compounds in soil depends on physiochemical properties of soil, as well as the solubility and speciation of sulfonamide antibiotics. Thus, due to higher water solubility of SMZ (1500 mg L^−1^), it has higher leaching capability [37]. The higher mobility and transport of SMZ in porous media has also been reported by Chen et al. [35]. Zhao et al. [38] found higher concentrations of SMZ in manure amended soil, which decreased over time. The faster release and dissipation of SMZ from manure resulted in higher leaching in the beginning [39]. Pan and Chu [40] reported that sulfonamide antibiotics can be released and leached into deeper soil layers and even groundwater through infiltration. However, the higher water solubility and shortened half-life of sulfonamide antibiotics results in quicker release and transport [41]. Moreover, some other biotic and abiotic factors influence the dissipation of sulfonamide antibiotics in soil too. For instance, the presence of resistant bacteria in manure could degrade antibiotics and decease their release over time [42]. Additionally, the infiltration and subsurface interflow may hasten the transport and leaching of SMZ through soil. Therefore, the release of SMZ from manure and its quicker transport could result in contamination of the adjacent water bodies, underground water, and edible crops, subsequently posing serious risk for ecosystem. Several studies reported that manure serves as an antibiotics reservoir [43]. However, the transport and leaching of SMZ from contaminated manure can be lessened with the application of BC into soil. Our results exhibited the highest desorption and leaching of SMZ in all collected leachates in soil S3 as it was amended with manure containing the highest SMZ concentration (55 mg kg^−1^). However, when amended with 2.0% BC, the SMZ leaching was reduced to more than twofold. Thus, our results suggested a quicker desorption of SMZ from manure and its leaching through soil during the initial 40 h, which gradually reduced over time. Further, the addition of BC resulted in significant decline in desorption and leaching of SMZ from contaminated manure.

### 2.4. Leaching Kinetics of SMZ

**Figure 4:**
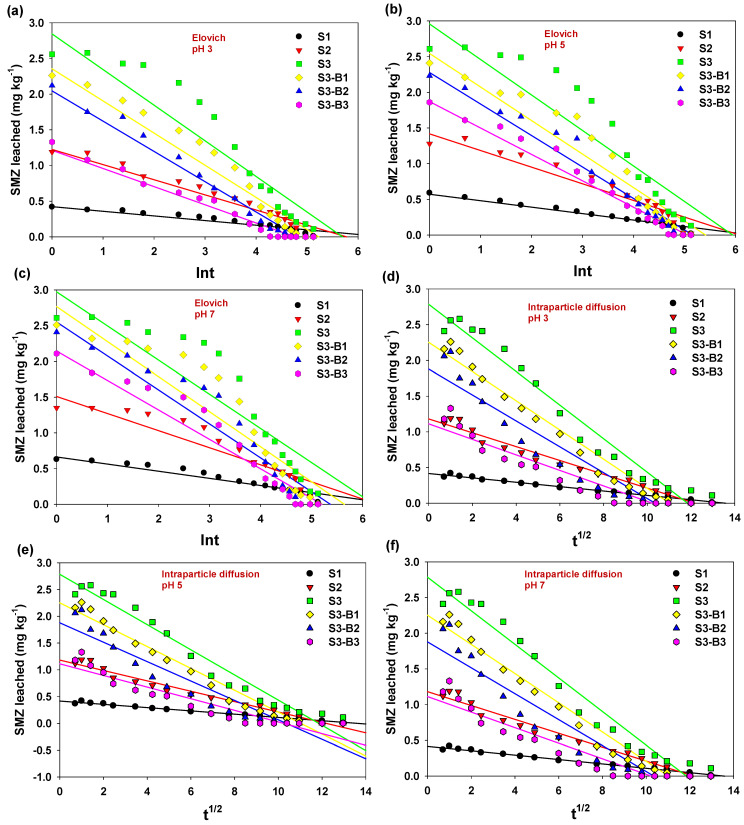
The Elovich (**a**–**c**) and intraparticle diffusion kinetic model plots (**d**–**f**) for sulfamethoxazole (SMZ) leaching from contaminated manure-amended soils with and without biochar applications at pH of 3.0, 5.0, and 7.0 (S1: soil with manure containing 13.75 mg kg^−1^ of SMZ, S2: soil with manure containing 27.5 mg kg^−1^ of SMZ, S3: soil with manure containing 55 mg kg^−1^ of SMZ, S3-B1: treatment S3 with 0.5% biochar application, S3-B2: treatment S3 with 1.0% biochar application, S3-B3: treatment S3 with 2.0% biochar application).

The leaching data for SMZ transport through contaminated-manure-amended soil with and without BC application at pH 3.0, 5.0, and 7.0 were simulated with kinetic models. The calculated coefficients of determination (*R*^2^) for the applied kinetics models are shown in Table 2. It was seen that intraparticle diffusion and Elovich models were the best fitted to leaching kinetics data (*R*^2^ = 0.88–0.99 and 0.81–0.98, respectively), whereas, the first order model was marginally fitted (*R*^2^ = 0.78–0.95). The plots of the Elovich model for SMZ leaching at pH 3.0, 5.0, and 7.0 are shown in Figure 4a, Figure 4b and Figure 4c, respectively, while the plots for intraparticle diffusion at pH 3.0, 5.0, and 7.0 are shown Figure 4d, Figure 4e and Figure 4f, respectively. Overall, the fitness of the kinetics models to the SMZ leaching data was, in order, intraparticle diffusion > Elovich > first order > pseudo-second order ≥ second order > power function. Table 3 shows the predicted parameters obtained from the kinetics leaching data. The Elovich model predicted leaching rate constant (β) at pH 3.0 was the lowest in S1 (0.424), which increased by 2.8-fold in S2 (1.225), and 6.7-fold in S3 (2.844), indicating a direct link of SMZ concentration in manure with its leaching in soil. However, the predicted β at pH 3.0 after the application of BC at 0.5%, 1.0%, and 2.0% was 2.355, 2.046, and 1.213, respectively, which was 21%, 39%, and 134%, respectively, higher that the same treatment without BC application (S3). A similar trend was also seen at pH 5.0 and 7.0, depicting the highest leaching in S3, and reduced leaching with BC application; however, the predicted β values for a specific treatment were higher at pH 7.0, followed by pH 5.0, and pH 3.0, suggesting a higher leaching rate of SMZ at pH 7.0. Similar to β, the intraparticle diffusion model predicted rate constant (c) at pH 3.0 was lower in S1 (0.416), which significantly increased in S2 (1.181), and S3 (2.784). However, the addition of BC resulted in reduced c values in S3-B1, S3-B2, and S3-B3 (2.252, 1.8778, and 1.112, respectively). This trend of intraparticle diffusion model predicted c variations at different pH levels following the same trend exhibited by the Elovich model-predicted β. 

The fitness of the intraparticle diffusion and Elovich models to the SMZ leaching data indicated the involvement of pore diffusion and chemical process in transport of SMZ through soil columns. Therefore, the release of SMZ from the contaminated manure and its leaching within the soil columns could be due to surface reactions and diffusion [44]. Diffusion into intra-aggregate pores and organic matter could be responsible for determining the leaching and retention of sulfonamide antibiotics [45]. Scheib [46] has also reported a quicker diffusion of sulfonamide antibiotic compounds into soil pores. However, the physiochemical properties, prevailing pH, and amount of organic matter affect the leaching as well [47]. Therefore, the higher leaching and mobility of SMZ in soil could be dangerous to the ecosystem. Förster et al. [48] stated that quickly dissipating sulfonamide antibiotics are highly mobile in soil, and are capable of leaching into deeper soil layers. Additionally, the lateral mobility of sulfonamide antibiotics through diffusion can potently pollute nearby waterbodies [39]. Therefore, long term application of SMZ-laden manure to soil can result in substantially higher levels of SMZ residues in the environment.

### 2.5. Significance of Biochar in Reducing SMZ Leaching 

**Figure 5:**
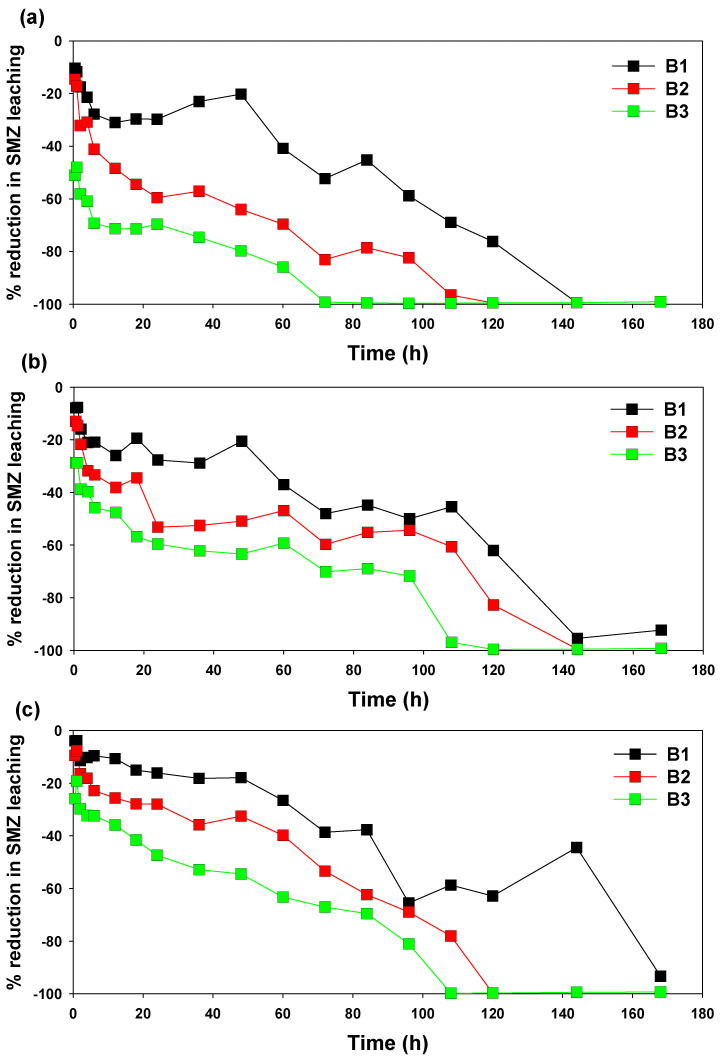
Percent reduction in sulfamethoxazole (SMZ) leaching at pH 3.0 (**a**), pH 5.0 (**b**), and pH 7.0 (**c**) from soil amended with manure containing 55 mg kg^−1^ of SMZ after biochar applied at 0.5% (B1), 1.0% (B2), 2.0% (B3) application rate.

The impacts of BC application on retention and leaching of SMZ released from contaminated manure were investigated at pH 3.0, 5.0, and 7.0, and the results are presented in Figure 5. Compared with soil S3, the highest percent reduction in SMZ leaching during all time intervals was demonstrated with 2.0% BC application, followed by 1.0% (B2) and 0.5% (B1) BC application. Comparing the percent reduction at different pH levels, the highest reduction was observed at pH 3.0, suggesting higher retention and adsorption of SMZ at this pH level. At pH 3.0, B3 was able to reduce SMZ leaching to 99% after 72 h, while B2, and B1 reached 99% leaching reduction after 120 and 144 h, respectively, as compared to the same treatments without BC application (S3). The time to reach 99% reduction in SMZ leaching was delayed when pH was increased, which was 120 h for B3, and 144 h for B2, while B1 did not reach to 99% reduction even after 168 h. These results demonstrate the significance of BC application in reducing SMZ leaching from soil, which could be of critical importance for ecosystem health. 

The potential of BC for adsorbing organic pollutants such as phenols, phenanthrene, polychlorinated biphenyls, and polycyclic aromatic hydrocarbons has been reported previously, while little is known about its adsorption efficiency for antibiotics [49]. Our results demonstrated a higher efficiency of jujube wood waste derived BC for enhancing SMZ retention in soil and reducing its leaching, which subsequently could lessen the contamination of groundwater, and could improve physiochemical characteristics of soil. Previously, Yao et al. [50] demonstrated the potential of BC for reducing the leaching of pharmaceutical products such as SMZ from soil columns. They reported that the transport and mobility of SMZ was substantially reduced after BC application to the soil. Likewise, Srinivasan and Sarmah [51] and Vithanage et al. [52] observed higher retention of SMZ antibiotics in soil when amended with BC. The reduction in SMZ leaching with the application of BC could be attributed to higher adsorption of SMZ onto the matrix of BC through multiple mechanisms. The variation in SMZ leaching with changing pH suggested the involvement of electrostatic interactions on SMZ adsorption. The higher retention at pH 3.0 was the result of electrostatic attractive forces between positively charged SMZ, and negatively charged biochar and soil surfaces. Additionally, π-π EDA interactions and H-bonding could also be involved in SMZ adsorption onto graphitized structured BC with O-containing functional groups [53]. The SMZ molecule behaves as a strong electron acceptor owing to presence of N-heteroaromatic rings and amino functional groups, while, due to the presence of O–H and C–H functional groups (as shown in Figure 1d), BC can serve as electron donor [54,55]. Therefore, the transfer of electrons from surface functional groups of BC to the SMZ molecule resulted in the generation of stronger π-π EDA interactions. On the other hand, Lewis acid-base electron interaction may help the adsorption of SMZ zwitterion onto biochar [56]. Various researchers have reported previously that π-π EDA interactions and H-bonding were the major mechanisms for SMZ adsorption onto BC due to carbonized and graphitized structure of BC [39,51]. Therefore, the reduced leaching of SMZ through BC amended and un-amended soil was due to retention and adsorption SMZ onto BC via multiple mechanisms. Moreover, the retention of SMZ in soil increased with increasing BC application rate, depicting a stronger effect of BC in reduction of SMZ leaching. Thus, jujube wood waste-derived BC could be used as a cost-effective, environment friendly, efficient, and green technology for reducing the leaching of SMZ in soil amended with SMZ-laden manure.

## 3. Materials and Methods

### 3.1. Biochar Production and Characterization 

Jujube wood waste was collected from Agriculture Research Farm Derab, King Saud University, Riyadh. The collected waste was washed, dried in air, and cut into small pieces. A known mass of the jujube wood waste was pyrolyzed in a digital muffle furnace (Wisetherm FH14, Wertheim, Germany) at 500 °C for 240 min under limited oxygen supply. The obtained BC was washed with deionized water, dried in an oven, ground, and stored. 

The produced BC was characterized for chemical, physical, and structural properties. The proximate analyses (moisture, volatile matter, ash, and total solids) were conducted by following the standard procedures of ASTM 123 D1762-84 [57]. The EC and pH were determined in 1:10 suspension in deionized water, while the CEC was determined by using standard protocol [58]. The surface morphology of BC was observed by using an electron microscope (SEM, EFI S50 Inspect, Eindhoven, The Netherlands), while the mineralogical composition was studied by using X-Ray diffractometer (MAXima X XRD-7000, Shimadzu, Kyoto, Japan), respectively. The composition of the surface functional groups was investigated by FTIR (FTIR: Bruker Alpha-Eco ATR-FTIR, Bruker Optics, Inc. Germany).

### 3.2. Soil and Manure Sample Collection and Characterization

Soil and manure samples were collected from Al-Kharj (24°8′54′′ N and 47°18′18′′ E), Saudi Arabia, which is known for its agricultural and dairy products. Composite soil sample was collected from a depth of 0–30 cm with the help of auger. Approximately 1.0 kg of composite manure sample was collected in acetone rinsed plastic container from a dairy farm. Multiple samples of soil and manure were collected and composite samples were prepared. All samples were kept in an icebox and transferred to the laboratory for further analyses.

The pH and EC of soil was measured in 1.25 ratio suspension in deionized water, whereas, the pH and EC or manure samples were measured in 1:10 ratio suspension in deionized water [59]. The organic matter was determined by following the Walkley–Black method [60]. Available P was extracted with the Olsen method and analyzed on a spectrophotometer [61]. Likewise, K, Na, CaCO_3_, and nitrate (NO_3_^−^) in soil and manure samples were analyzed by following the standard protocols [62]. The particle size distribution of soil samples was examined with a Malvern Mastersizer 2000 [63] whereas, the proximate analyses of manure samples were determined by ASTM D1762-84 standard methods [57]. 

### 3.3. Manure Spiking and Leaching Trials

**Scheme 1:**
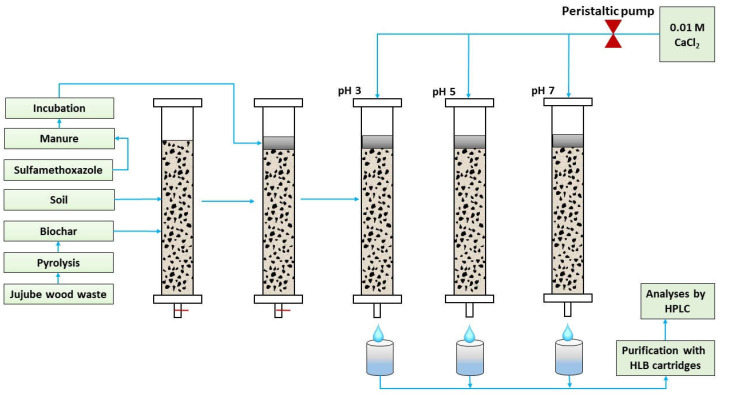
Schematic illustration for the experimental set-up used to investigate the sulfamethoxazole leaching from soil columns.

The analytical grade SMZ with 98% purity was purchased from Sigma-Aldrich Co., Steinheim, Germany. The SMZ was added into the freshly collected manure at the rate of 3.5 g kg^−1^ and kept in the dark at room temperature (23 ± 2 °C) for 2 weeks. Thereafter, the collected soil with particle size of <2 mm, was mixed thoroughly with 0.0%, (S), 0.5% (B1), 1.0% (B2), and 2.0% (B3) of BC and filled into PVA columns of 6.0 cm internal diameter and 40 cm height (Scheme 1). The bottoms of the columns were closed with a porous membrane and filter paper. The soil was filled by gently taping the columns to ensure a density of 1.48 g cm^−3^ to mimic with natural condition in the field. The surface layer of soil in the column was mixed with specific amount of SMZ-spiked manure. Specifically, the soil without BC applications (S) was amended with spiked manure to maintain SMZ concentrations of 13.75 mg kg^−1^ (S1) 27.5 mg kg^−1^ (S2), and 55 mg kg^−1^ (S3), whereas, the soils with BC applications (B1, B2, and B3) were amended with spiked-manure to acquire 55 mg kg^−1^ of SMZ to make the S3-B1, S3-B2, and S3-B3 treatments. The columns were wrapped with a black sheet to avoid photodegradation of SMZ. Thereafter, the columns were irrigated to their field capacity using deionized water for 5 days to acquire equilibrium stage. After equilibrating, glass beads were spread on the surface of the soil in column and 0.01 M CaCl_2_ solutions with initial pH of 3.0, 5.0, and 7.0 were passed with a flow rate of 10 mL h^−1^. The flow rate of the background solution was maintained with the help of a peristaltic pump. Leachates were collected at different time intervals, passed through 0.2 µm syringe filters and stored at −10 °C for further analyses. 

### 3.4. Solid-Phase Extraction of SMZ

The HLB cartridges (3 mL/60 mg) were used for solid-phase extraction (SPE) and clean-up of SMZ antibiotics. Initially, 3.0 mL of methanol, 3.0 mL of 0.5 N HCl, and 3.0 mL of HPLC grade water were used to activate HLB cartridges. Thereafter, the collected leachates were passed through the cartridges by using 40 psi of vacuum with a flow rate of ~2 mL min^−1^. After passing all the leachate, the cartridges were rinsed with 3.0 mL HPLC grade water and dried for 30 min in vacuum. Then, SMZ was eluted with 2.5 mL of methanol into a collection vial, and mixed on a vortex mixer. The methanol from the collection vials was evaporated to 150 µL by gentle heating at 30 °C under N_2_ atmosphere and 250 µL of mobile phase (20% methanol + 80% phosphoric acid) was added. These extracts were then passed through 0.45 µm syringe filters and added into amber-colored HPLC vials for analyses. All the samples were prepared and extracted three times including blanks. All samples, including blanks, were prepared and extracted three times.

### 3.5. Quantification of SMZ

The concentrations of SMZ in the prepared and cleaned-up extracts were analyzed by using high-performance liquid chromatography (HPLC; Prominence-i, LC-2030C, Shimadzu, Kyoto, Japan), with a reversed-phase Raptor C18 column (100 mm × 21 mm, 2.7 μm particle size) and PDA detector. Acetonitrile and phosphoric acid (pH = 3.0) were used as mobile phase A and B, respectively. The mobile phase A was maintained at 93% for an initial 5.0 min, then changed to 70% from 5.0–18 min, and held at 70% till 25 min. Then, the phosphoric acid: acetonitrile ratio was changed to 93:7.0 from 25–30 min and held up to 35 min. A calibration curve (*R*^2^ > 0.99) was constructed between SMZ concentrations in standards versus absorbance (%) by the HPLC. The standards were also fed as unknown samples for quality assurance. The samples with known concentrations of SMZ were used to estimate percent recovery, which was found to be 67.47 ± 4.55%.

### 3.6. Kinetics Modeling 

Adsorption, desorption and leaching are time-dependent processes. The sorption of adsorbate from a bulk solution phase to adsorbent surface is significantly influenced by the sorption rate. The sorption could be owing to the mass transfer or diffusion. Thus, investigating the kinetics of adsorbate sorption onto adsorbent is of critical importance to investigate the release and retention of an adsorbate. Therefore, the dynamics of SMZ leaching from soil columns were explored by simulating the leaching data with kinetic models as shown in Equations (1)–(6) [64]. Various kinetics models such as first order, second order, pseudo-second order, Elovich, power function, and intraparticle diffusion can be used to investigate the sorption and leaching of adsorbate from adsorbent.

(1) $$\text{First-order}\;lnq_{t}=lnq_{o}-k_{1}t$$

(2) $$\text{Second-order}\;\frac{1}{q_{t}}=\frac{1}{q_{o}}-k_{2}t$$

(3) $$\text{Pseudo-second-order}\;\frac{t}{q_{t}}=\frac{1}{k_{2}^{\prime }q_{e}^{2}}+\frac{1}{q_{e}}t$$

(4) $$\mathrm{Elovich}\;q_{t}=\frac{1}{\beta }ln\;\left(\alpha \beta \right)+\frac{1}{\beta }\;lnt$$

(5) $$\mathrm{Power}\;\mathrm{function}\;lnq_{t}=lnb+k_{f}\left(lnt\right)$$

(6) $$\mathrm{Intraparticle}\;\mathrm{diffusion}\;q_{t}=c+k_{id}t^{0.5}$$

where *t* is time, $q_{t}$ is the concentration of SMZ leached at time *t* (mg kg^−1^), and $q_{o}$ is the concentration of SMZ leached at time 0 (mg kg^−1^). $k_{1}\;$and $k_{2}\;$are first and second-order rate constant, respectively, and $q_{e}\;$is the amount of SMZ leached at equilibrium (mg kg^−1^). $k_{2\;}^{\prime }\;$is the rate constant for pseudo-second-order, *α* is initial sorption rate (mg kg^−1^ min^−1^), *β* is sorption constant, $k_{f}\;$is rate coefficient (mg kg^−1^ min^−1^), *b* is constant, *c* is diffusion constant, and $k_{id}$ is apparent diffusion rate constant ([mg kg^−1^]^−0.5^).

## 4. Conclusions

Leaching behavior of sulfamethoxazole (SMZ) through contaminated-manure-amended sandy loam soil at different pH levels was investigated in this study. The significance of jujube wood waste-derived biochar (BC) to enhance SMZ retention in soil was evaluated. The highest SMZ leaching was observed at pH 7.0, which reduced with lowering pH owing to the variation in SMZ speciation. During the initial 40 h, the release of transportation of SMZ was rapid, which gradually reduced with the passage of time. The highest SMZ was leached through soil amended with manure spiked at higher SMZ concentration (55 mg kg^−1^). Application of BC significantly decreased the leaching of SMZ through sandy loam soil. BC applied at 2.0% resulted in the highest SMZ retention (>2-folds) as compared to the same treatment without BC application. Our results depicted the substantial contribution of prevailing pH, SMZ concentration in applied manure, and the amount of BC applied in SMZ retention and leaching in a sandy loam soil. Electrostatic interactions, H-bonding, and π-π electron donor acceptor interactions could be the possible mechanisms responsible for higher SMZ adsorption onto BC. Therefore, we concluded that long-term application of SMZ-laden manure could potentially contaminate ground and surface water resources due to the higher mobility of SMZ. Jujube wood waste-derived BC could serve as an efficient and cost-effective technology to reduce the mobility of SMZ in contaminated-manure amended soil.

## Figures and Tables

**Table 1:** Selected physiochemical properties and proximate attributes soil, manure, jujube wood waste (FS) and FS-derived biochar (BC) used in this study.

Property	Unit	Soil	Manure	FS	BC
Sand	%	63.19	--	--	--
Silt	%	33.41	--	--	--
Clay	%	3.39	--	--	--
Texture	--	Sandy loam	--	--	--
pH	--	7.21	7.72	5.42	9.56
EC	dS m^−1^	2.41	6.61	0.77	0.57
Organic matter	%	0.62	10.34	--	--
CEC	cmol kg^−1^	21.19	57.29	64.93	25.78
CaCO_3_	%	8.45	--	--	--
Available P	mg kg^−1^	9.16	1970.82	63.81	77.94
Nitrate	mg kg^−1^	0.88	13.9	--	--
Available K	mg kg^−1^	78.00	40.16	--	--
Available Na	mg kg^−1^	25.81	46.09	--	--
Yield	%	--	--	--	36.7
Moisture	%	1.62	13.03	6.16	2.88
Volatiles	%	--	38.72	60.03	23.4
Fixed carbon	%	--	19.29	29.14	62.68
Ash	%	--	28.96	4.67	11.02

**Table 2:** The calculated coefficient of determination (*R*^2^) of kinetic models for sulfamethoxazole leaching from various contaminated manure-amended soils with and without biochar applications at pH 3, 5, and 7 (S1: soil with manure containing 13.75 mg kg^−1^ of SMZ, S2: soil with manure containing 27.5 mg kg^−1^ of SMZ, S3: soil with manure containing 55 mg kg^−1^ of SMZ, S3-B1: treatment S3 with 0.5% biochar application, S3-B2: treatment S3 with 1.0% biochar application, S3-B3: treatment S3 with 2.0% biochar application).

Sorbent	First Order	Second Order	Pseudo-Second Order	Elovich	Intraparticle Diffusion	Power Function
pH 3						
S1	0.88	0.74	0.44	0.89	0.99	0.43
S2	0.78	0.38	0.38	0.93	0.97	0.53
S3	0.88	0.88	0.88	0.87	0.96	0.50
S3-B1	0.90	0.48	0.48	0.94	0.96	0.59
S3-B2	0.94	0.61	0.61	0.98	0.89	0.66
S3-B3	0.89	0.83	0.83	0.97	0.88	0.66
pH 5						
S1	0.87	0.46	0.46	0.95	0.95	0.48
S2	0.89	0.55	0.55	0.90	0.99	0.48
S3	0.88	0.85	0.85	0.85	0.99	0.47
S3-B1	0.94	0.53	0.53	0.92	0.97	0.56
S3-B2	0.87	0.47	0.47	0.96	0.97	0.61
S3-B3	0.90	0.61	0.61	0.97	0.94	0.62
pH 7						
S1	0.89	0.50	0.50	0.91	0.94	0.43
S2	0.78	0.31	0.31	0.86	0.98	0.44
S3	0.88	0.81	0.81	0.81	0.97	0.43
S3-B1	0.95	0.38	0.64	0.86	0.97	0.50
S3-B2	0.86	0.58	0.58	0.90	0.97	0.55
S3-B3	0.87	0.67	0.67	0.91	0.96	0.58

**Table 3:** Parameters derived from the kinetic models for sulfamethoxazole leaching from various contaminated manure-amended soils with and without biochar applications at pH 3, 5, and 7 (S1: soil with manure containing 13.75 mg kg^−1^ of SMZ, S2: soil with manure containing 27.5 mg kg^−1^ of SMZ, S3: soil with manure containing 55 mg kg^−1^ of SMZ, S3-B1: treatment S3 with 0.5% biochar application, S3-B2: treatment S3 with 1.0% biochar application, S3-B3: treatment S3 with 2.0% biochar application).

Sorbent	First Order	Second Order	Pseudo-Second Order			Elovich		Intraparticle Diffusion		Power Function	
	*k*_1_	*k*_2_	*k*_2_^′^	*q_e_*	*h*	*a*	*β*	*k_id_*	*c*	*k_f_*	*b*
pH 3											
S1	−0.016	0.278	−0.009	7.195	−0.464	−0.065	0.424	−0.031	0.416	−0.065	0.424
S2	−0.032	2.763	−0.046	0.724	−0.024	−0.212	1.225	−0.097	1.181	−0.212	1.225
S3	−0.020	0.041	−0.003	48.283	−7.321	−0.499	2.844	−0.235	2.784	−0.499	2.844
S3-B1	−0.042	4.148	−0.073	0.482	−0.017	−0.450	2.355	−0.204	2.252	−0.450	2.355
S3-B2	−0.049	5.545	−0.115	0.361	−0.015	−0.424	2.046	−0.181	1.878	−0.424	2.046
S3-B3	−0.054	7.660	−0.270	0.261	−0.018	−0.254	1.213	−0.109	1.112	−0.254	1.213
pH 5											
S1	−0.014	0.140	−0.010	14.312	−2.020	−0.090	0.571	−0.040	0.546	−0.090	0.571
S2	−0.021	0.183	−0.004	10.924	−0.505	−0.233	1.417	−0.109	1.387	−0.233	1.417
S3	−0.018	0.033	−0.003	59.935	−10.592	−0.497	2.957	−0.238	2.924	−0.497	2.957
S3-B1	−0.031	0.430	−0.008	4.647	−0.181	−0.470	2.544	−0.217	2.459	−0.470	2.544
S3-B2	−0.041	4.142	−0.073	0.483	−0.017	−0.441	2.277	−0.195	2.146	−0.441	2.277
S3-B3	−0.048	5.533	−0.114	0.361	−0.015	−0.370	1.870	−0.163	1.755	−0.370	1.870
pH 7											
S1	−0.013	0.096	−0.018	20.797	−7.741	−0.100	0.663	−0.046	0.647	−0.100	0.663
S2	−0.022	0.482	−0.008	4.147	−0.144	−0.239	1.510	−0.115	1.496	−0.239	1.510
S3	−0.018	0.031	−0.002	65.256	−10.339	−0.478	2.974	−0.234	2.972	−0.478	2.974
S3-B1	−0.028	0.266	−0.005	7.521	−0.281	−0.490	2.767	−0.234	2.727	−0.490	2.767
S3-B2	−0.048	5.435	−0.110	0.368	−0.015	−0.475	2.551	−0.221	2.475	−0.475	2.551
S3-B3	−0.052	6.497	−0.152	0.308	−0.014	−0.412	2.144	−0.189	2.063	-0.412	2.144

## Data Availability

Not applicable.

## Sample Availability

Samples of the compounds are not available from the authors.
